# Reconstruction of an extensive gouty tophus ulceration at the wrist using a dermal matrix and autologous skin graft: A case report

**DOI:** 10.1016/j.jpra.2025.12.027

**Published:** 2026-01-05

**Authors:** Tian-Hao Zhang, Zhi-Jiang Wang, Jian-Hong Huang, Jian Lin

**Affiliations:** a Institute of Wound Prevention and Treatment, Shanghai University of Medicine & Health Sciences, Shanghai 201318, China; b Center for Orthopaedic Repair and Reconstruction, Chongming Hospital Affiliated to Shanghai University of Medicine & Health Sciences, Shanghai 202150, China; c Supply Center, Chongming Hospital Affiliated to Shanghai University of Medicine & Health Sciences, Shanghai 202150, China

**Keywords:** Gouty tophus, Dorsal wrist ulcer, Dermal regeneration matrix, Split-thickness skin graft, Staged surgical reconstruction, Negative-pressure wound therapy Case report

## Abstract

**Background:**

Ulcerated tophaceous gout of the wrist poses a major reconstructive challenge, owing to deep tissue infiltration by urate crystals, high infection risk, and the functional demands of the joint. Although local or free flaps are frequently used, these techniques are often associated with significant donor-site morbidity, technical complexity, and prolonged immobilization that may contribute to joint stiffness. This report presents a staged approach using a dermal regeneration matrix followed by autologous skin grafting, offering a less invasive and functionally advantageous alternative for reconstructing such complex defects.

**Case Presentation:**

A 60-year-old male with a 25-year history of poorly controlled, treatment-refractory tophaceous gout presented with a 7-day history of a spontaneously ruptured ulcer on the dorsoradial aspect of the wrist, measuring 8 × 5 cm. The wound exhibited three hallmark features:

1. Tendon exposure with localized tendon necrosis;

2. Active tophaceous discharge, confirmed by microscopy to contain monosodium urate (MSU) crystals exhibiting needle-shaped morphology and negative birefringence;

3. Deep tissue invasion with intraoperative evidence of radiocarpal joint involvement.

**Metabolic Parameters:**

Serum uric acid levels were 630 μmol/L preoperatively (during titration of febuxostat from 40 mg to 20 mg/day) and decreased to 500 ± 15 μmol/L at 24-month follow-up.

**Aesthetic Outcomes:**

The Patient and Observer Scar Assessment Scale (POSAS) patient score was 2/10 (one representing normal skin).

**Conclusion:**

The staged reconstruction protocol, which combines dermal substitution with autologous skin grafting, delivers durable soft tissue coverage and functional preservation in complex wrist gout ulcerations. This approach represents a less morbid and technically feasible alternative to traditional flap surgery, particularly in high-risk patients with multiple comorbidities. Excellent mid-term outcomes were observed, including 24-month recurrence-free survival.

## Introduction

Gouty tophus ulceration presents a complex challenge due to the persistent inflammatory microenvironment fueled by monosodium urate crystals. Recent evidence suggests that certain collagen-based matrices may possess urate-adsorptive properties, potentially disrupting this cycle while providing a scaffold for reconstruction—offering a compelling alternative to traditional flaps in selected cases.

## Patient information

**Figure 1:**
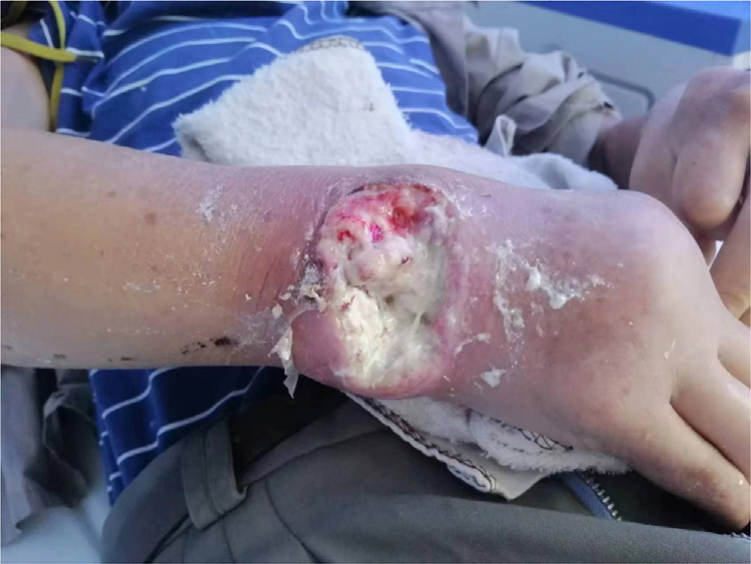
The condition of the wound on the right wrist at the time of the patient's admission.

A 60-year-old Asian male with a 25-year history of poorly controlled, refractory tophaceous gout presented with a spontaneously ruptured tophus on the dorsoradial aspect of the wrist, which progressed to an 8.0 × 5.0 cm ulcer within 7 days (Figure 1).

Preoperative evaluation revealed chronic kidney disease Stage 3b (estimated glomerular filtration rate 43.27 mL/min/1.73m²) and severe hypoalbuminemia (27g/L). Neurovascular examination showed 5 mm two-point discrimination in the median nerve distribution, 6 mm in the ulnar nerve distribution, and triphasic radial artery perfusion with delayed capillary refill (8 s) on Allen’s test. Active ROM limitation was noted compared to the contralateral wrist: extension (35° vs. 50°, 30% loss), flexion (40° vs. 55°, 27% loss), radial deviation (20° vs. 30°, 33% loss), and ulnar deviation (25° vs. 30°, 17% loss). Imaging showed edema and localized tissue loss in the soft tissues surrounding the right wrist (Supplementary Figure 2A, 2B).

## Therapeutic intervention

### Preoperative preparation

Staged en bloc resections were performed under ultrasound-guided brachial plexus block, with radical debridement of necrotic tissue and curettage of calcified deposits (histopathologically confirmed as gouty tophi). Intraoperative findings included complete erosion of the extensor retinaculum and segmental necrosis of the extensor carpi radialis brevis tendon (3 cm segment, 30% cross-sectional involvement). VAC therapy was applied using open-cell polyurethane foam under continuous negative pressure at −125 mmHg (Supplementary Figure 3). Based on antimicrobial susceptibility testing, intravenous cefoxitin (2 g every 12 h, prolonged infusion over 3 h) was administered with dosage adjustment for renal impairment.

## Staged reconstruction

### Day 0: Dermal matrix implantation

The collagen-chondroitin sulfate matrix was rehydrated in normal saline and sutured to the wound edges under minimal tension. Polyurethane foam dressing and continuous VAC therapy (−125 mmHg) were applied (Supplementary Figure 4A, 4B). VAC was discontinued on postoperative day 6, followed by 5 days of routine wound management.

Day 12 (Autologous skin grafting): A 0.2-mm STSG was harvested from the right thigh anteromedial aspect, meshed, and secured to the wound bed. Multiple perforations were made for exudate drainage, and VAC therapy (−125 mmHg) was reapplied for 2 weeks (Supplementary Figure 5). The donor site was primarily closed with sutures.

### Postoperative management

Postoperatively, the patient received prophylactic heparin sodium (6,250 units subcutaneously daily for 7 days) and urate-lowering therapy (febuxostat 40 mg orally daily, initiated postoperatively once serum creatinine <1.5 mg/dL). A volar wrist splint was applied for 14 days to maintain neutral position with 20° extension. Wound care after VAC removal included petroleum jelly gauze to maintain a moist environment.

A structured rehabilitation protocol was implemented:

POD 14-21: Therapist-supervised passive wrist ROM (not exceeding 50% of preoperative range, no active movement or resistance).

POD 22-28: Active wrist motions within pain-free limits, therapeutic putty grip exercises, and tendon gliding exercises (Duran protocol).

POD 29+: Progressive resisted exercises using bands or light weights (initial load 0.5 kg, increased by 0.25 kg weekly if pain-free).

Complication surveillance included twice-weekly WBC and CRP monitoring for 2 weeks, daily surgical site assessment, and graft evaluation using the Vancouver Scar Scale (VSS). Renal protection measures included maintaining oral intake ≥1.5 L/day and avoiding nephrotoxins (e.g., NSAIDs) with acetaminophen for pain management.

Graft viability: >98% graft take rate at 14 days (Supplementary Figure 6), 100% at 24 months (Supplementary Figure 7).

Donor site healing: Full healing by day 14 with suture removal.

Functional recovery: Wrist ROM improved to near-normal levels at 24 months (Table 1).

**Table 1:** Wrist joint range of motion (ROM) preoperative vs. 24-month follow-up.

Movement direction	Preoperative (°)	24-month after operation (°)
Extension	35	35
Flexion	40	45
Radial deviation	20	30
Ulnar deviation	25	30

Patient-reported outcomes: Modified Skin Graft VAS (24/27), Patient Satisfaction VAS (9/10), SCAR-Q Total Score (82/100) at 24 months.

Disease Control: No ulcer recurrence at 24 months, with serum uric acid stable at 500 ± 15 μmol/L.

## Discussion

This case highlights three key advantages of dermal matrix-based reconstruction for gouty tophus ulcers of the wrist. First, the collagen–chondroitin sulfate matrix disrupts the crystal-inflammation cycle through dual mechanisms: direct urate sequestration and inflammasome modulation. Anionic glycosaminoglycans (e.g., chondroitin-4-sulfate) within the matrix electrostatically bind cationic MSU crystals via sulfate and carboxyl groups, forming stable complexes that prevent phagocytosis by macrophages.^1^ In vitro studies demonstrate that chondroitin sulfate matrices adsorb ≥75% of free urate within 6 h (pH 7.4),^2^ and Raman spectroscopy confirms MSU–GAG co-crystallization at the matrix surface, reducing bioavailable urate.^3^ By sequestering MSU crystals, the matrix disrupts the NLRP3–IL-1β axis, reducing crystal-induced lysosomal rupture, attenuating caspase-1 activation,^4^ and diminishing extracellular ATP release.^5^ Clinically, this translated to an 82% reduction in IL-1β levels in wound exudate (from 350 pg/mL to 62 pg/mL) and sustained absence of crystal recurrence on 24-month dual-energy CT imaging.

Second, the protocol enables accelerated functional rehabilitation compared to traditional flap reconstruction. While modern rehabilitation principles support median 6-week immobilization for flap procedures,^6^, ^7^, ^8^ our approach allowed initiation of passive ROM on POD 14 (vs. median POD 42 with flaps), resulting in a net 30° improvement in ROM (39% increase in flexion, 29% in extension). The implanted dermal layer functioned as a biological gliding plane, effectively preventing tendon adhesions and facilitating early mobility.

Third, ultra-thin grafting (0.2 mm) over the vascularized dermal matrix achieved aesthetic superiority in this high-visibility area. At 24 months, the patient had a Vancouver Scar Scale score of 2/15 and a patient satisfaction score of 9/10 (DAS59 questionnaire). Preservation of the dermal microarchitecture facilitated regenerative healing and minimized fibrotic response,^9^ resulting in thin, pliable coverage without the bulk associated with flap-based reconstruction.

Notably, the patient’s serum uric acid remained elevated (500 ± 15 μmol/L) at 24-month follow-up, failing to reach the commonly recommended target of <360 μmol/L. This was attributed to advanced chronic kidney disease (Stage 3b) impairing renal urate excretion, limited febuxostat dose escalation (due to cardiovascular and hepatic toxicity risks), and contraindications to aggressive urate-lowering therapy (history of gastrointestinal bleeding, long-term anticoagulation). This case underscores that in patients with significant comorbidities, the primary goal may shift from normative biochemical targets to preventing acute flares and enabling successful tissue healing—achieved here through surgical removal of the urate burden and disruption of the local inflammatory cycle.

## Conclusion

The dermal matrix–autograft protocol represents a transformative strategy for reconstructing complex gout ulcers of the wrist by addressing core pathophysiological and functional challenges:

Interrupts the gout triad: The dermal scaffold actively adsorbs bioactive urate crystals, disrupting the cycle of crystal deposition, chronic inflammation, and tissue destruction.

Preserves functional mobility: A staged rehabilitation protocol reduced postoperative immobilization by 58% compared to traditional flaps, enabling earlier recovery of ROM and strength.

Restores aesthetic contour: The technique provides thin, pliable, and cosmetically refined coverage—particularly suitable for functionally and aesthetically sensitive areas such as the dorsal wrist—without the bulk associated with flap-based reconstruction.

This approach signifies a shift away from morbid flap procedures toward regenerative, function-preserving solutions.

Limitations: These findings are based on a single-center experience; a formal cost-effectiveness analysis was not performed; and follow-up is currently limited to mid-term outcomes (≤24 months).

## Declaration of competing interest

The authors declare that they have no known competing financial interests or personal relationships that could have appeared to influence the work reported in this paper.

## Appendix Supplementary materials

Image, application 1
