# Supplementary material for: Reconstruction of an extensive gouty tophus ulceration at the wrist using a dermal matrix and autologous skin graft: A case report
Source: JPRAS Open. 2026 Jan 5;48:706–10. doi: 10.1016/j.jpra.2025.12.027 (PMC12878680; doi:10.1016/j.jpra.2025.12.027)
Supplement: Supplementary file 1 [file mmc1.docx]

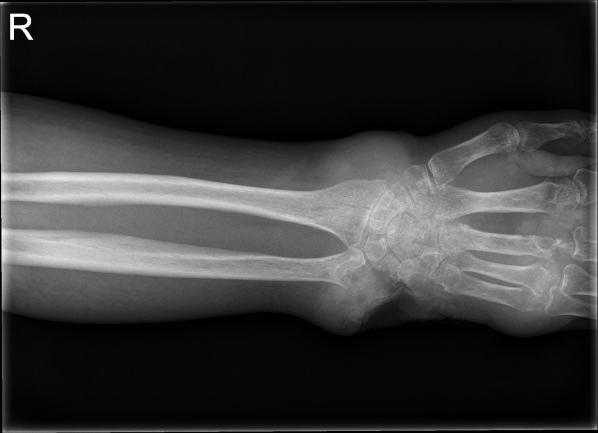

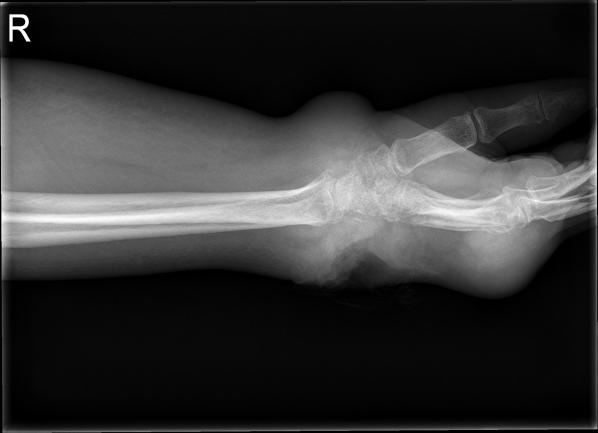


Fig.2A Fig.2B


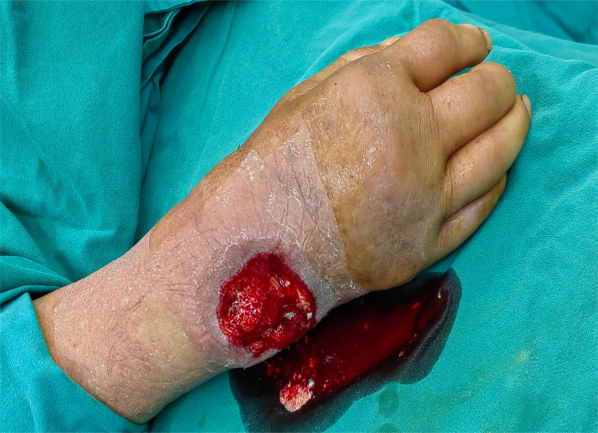


Fig.3


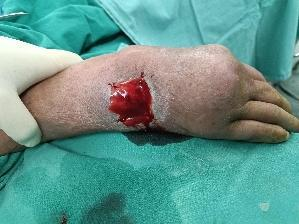

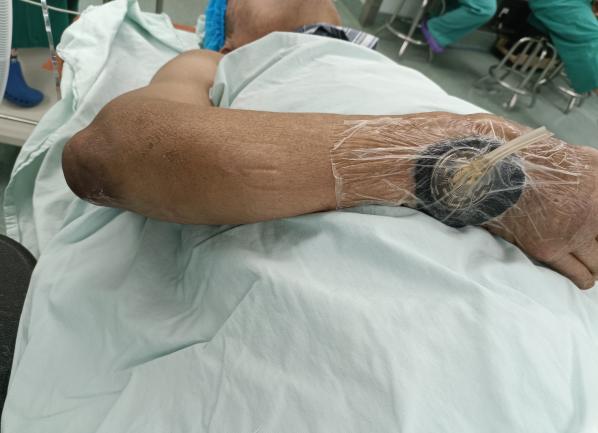


Fig.4A Fig.4B


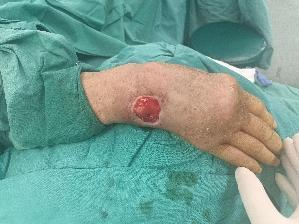


Fig.5


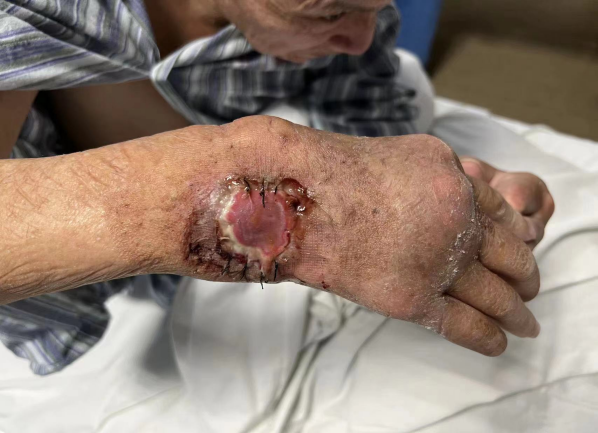


Fig.6


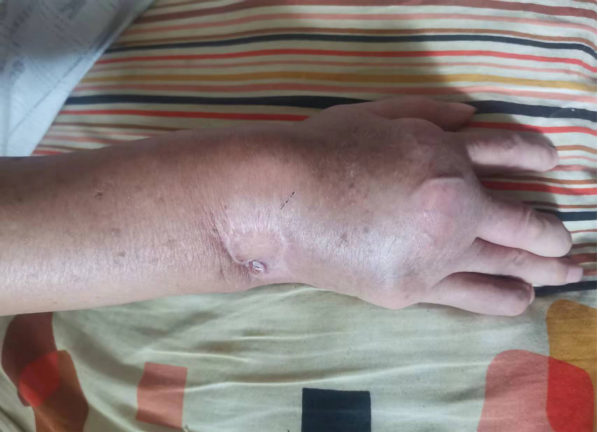


Fig.7

**Fig 2A.Right wrist AP radiograph at admission.**

**Fig 2B.Right wrist lateral radiograph on admission.**

**Fig 3.Condition of the wound after debridement.**

**Fig 4A.The wound was covered with artificial dermis.**

**Fig 4B.VAC therapy was applied to the wound.**

**Fig 5.Wound status post artificial dermis removal.**

**Fig 6.Autologous skin graft viability post-transplantation.**

**Fig 7.Right wrist wound status at 24-month follow-up.**
